# *Moringa oleifera* and Polyaluminum Chloride: Coagulant Combinations for Cyanobacteria Removal in Drinking Water

**DOI:** 10.1021/acsomega.5c03206

**Published:** 2025-08-22

**Authors:** Camila Laschiwitz Beghetto, Rúbia Martins Bernardes Ramos, Pablo Inocêncio Monteiro, Fatima de Jesus Bassetti, Lucila Adriani de Almeida Coral

**Affiliations:** 1 Posgraduate Program in Environmental Science and Technology, Federal University of Technology-Paraná (UTFPR), R. Deputado Heitor Alencar Furtado, 5000-CIC, Curitiba, PR CEP 81280-340, Brazil; 2 Posgraduate Program in Food Engineering, Chemical Engineering Department, 74354 Federal University of Parana, Curitiba, PR 81531-980, Brazil

## Abstract

Cyanobacteria represent a significant problem for water treatment systems due to their ability to form blooms, release harmful toxins, and decrease the efficiency of water treatment processes. This study investigated the efficacy of a natural coagulant and its combination with a chemical coagulant to remove cyanobacteria from water. The combination with natural coagulants reduces residual aluminum concentrations in the sludge and treated water and lowers process costs. A dosage of 20 mg L^–1^ of *Moringa oleifera* was identified as the most effective for the removal of turbidity (≈82%), color (67–76%), and cell density (88–97%). The proportion of 75% *Moringa oleifera* and 25% polyaluminum chloride was the optimal condition for cell removal (95%) and resulted in a low residual aluminum concentration in the treated water (0.030 mg L^–1^). The seasonal variation of cyanobacteria in the C/F/DAF treatment showed that 50% *Microcystis aeruginosa* and 50% *Cylindrospermopsis raciborskii* achieved the most effective removal of turbidity (94%) and cell density (≈99%). The color removal for this cell proportion was 80%. Based on the results, treatment efficiency was not affected by cell density or cell morphology. The analysis of dissolved organic carbon and total dissolved carbon showed no variation for most of the samples after C/F/DAF treatment. This study demonstrated the efficiency of combining coagulants, highlighting the potential of *Moringa oleifera* as a sustainable water treatment alternative.

## Graphical abstract

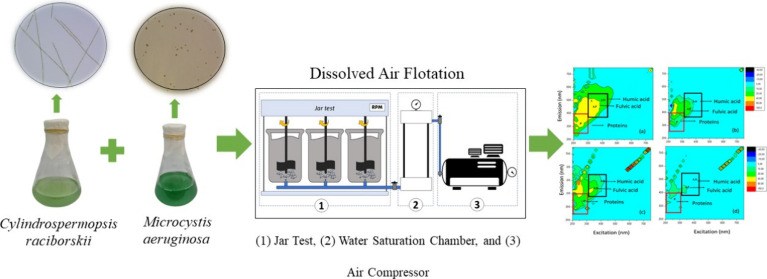

## 1 Introduction

Eutrophication, primarily driven by human activities, has been degrading water quality, promoting the proliferation of cyanobacteria.^1,2^ These microorganisms, such as *Cylindrospermopsis raciborskii* (*C. raciborskii*) and species of the genus *Microcystis*, exhibit high phenotypic plasticity^3,4^ and can form extensive blooms in water reservoirs. *Cylindrospermopsis raciborskii* (CR), also called *Raphidiopsis raciborskii*, is a filamentous cyanobacterium,^3,5^ whereas *Microcystis aeruginosai* (*M. aeruginosa*, MA) is a unicellular organism. Both CR and MA can synthesize cyanotoxins, and CR can fix nitrogen.^5^ Additionally, cyanobacterial blooms can cause serious harm to the population, potentially leading to the disruption of the water supply system^6^ and altering the taste and odor of the water.^7^ It can also release toxins into the water, posing public health risks, including liver, neurological, and digestive diseases.^8,9^

Coagulation is an important process in water treatment, contributing to the removal of impurities including cyanobacteria. However, inorganic coagulants such as aluminum chloride and aluminum sulfate have significant limitations, including the generation of nonbiodegradable sludge and high toxicity due to aluminum residues,^10^ which have been associated with neurodegenerative diseases. Polyaluminum chloride (PAC), although it presents a lower residual aluminum concentration compared to aluminum sulfate,^11^ can still compromise the safety of the treated water. Given these limitations, natural coagulants, such as *Moringa oleifera* (MO) seed extract, have been sought as they demonstrate high efficiency in removing cyanobacteria without the negative environmental impacts of traditional coagulants.

The moringa extract acts as a coagulant due to the presence of molecules that function as cationic polyelectrolytes.^12,13^ Additionally, when MO seeds are crushed and added to water, the proteins present attract microorganisms, clay, and other impurities^14,15^ The previous study^16^ mentions adsorption and neutralization as possible mechanisms of action for the coagulation of MO. Previous studies show that MO exhibits high efficiency in removing cyanobacteria^17,18^ and their toxins.^19,20^ The combination of MO with chemical coagulants, such as PAC, reinforces the advantage of improving the efficiency of removing impurities and harmful organisms. Additionally, it offers a way to lower costs and decrease the reliance on chemicals, leading to reduced negative impacts on both the environment and humans.^21^ Therefore, the use of natural coagulants, such as MO, along with PAC, can be an effective strategy to minimize environmental impacts and improve water and wastewater treatment processes. This approach helps reduce the residual organic matter associated with the exclusive use of MO and also decreases the dependence on chemical agents that may pose risks to human health.

This study investigates the combination of PAC with MO in water treatment using coagulation/flocculation (C/F) and dissolved air flotation (DAF) processes for cyanobacteria removal. C/F/DAF has proven to be a viable strategy for removing cyanobacteria, as it allows for the efficient separation of cells without inducing cell lysis, thereby reducing the release of toxins into the treated water.^22^ Since the performance of DAF depends on the coagulation/flocculation step,^23^ the combination of PAC and MO may promote the formation of flocs, optimizing the removal of organic matter and contaminants. Moreover, the association of two cyanobacterial species represents a condition closer to real contamination scenarios and contributes to a more comprehensive assessment of the treatment effectiveness. Here, water quality was assessed based on the reduction of turbidity, color, and cyanobacteria cell density. Thus, this work provides scientific information on the effectiveness of this integrated approach in improving water quality and minimizing the environmental and public health impacts associated with conventional treatments.

## 2 Materials and Methods

### 2.1 Materials

The Companhia de Saneamento do Paraná (Sanepar) provided PAC in a liquid form, with a basicity of 64.3% and an aluminum content ranging from 10.5 to 12.0%. The natural coagulant was obtained from *Moringa oleifera* seeds. The cyanobacteria used were the toxic strains of *M. aeruginosa* (BCCUSP232) and *C. raciborskii* (LP2). Sodium hydroxide (NaOH) and hydrochloric acid (HCl) were used to adjust the pH. Ultrapure water was used in all procedures.

### 2.2 Cultivation of *Microcystis aeruginosa* and *Cylindrospermopsis raciborskii*

The cell cultures of CR and MA were maintained in a controlled chamber under sterile conditions. The temperature was set to around 25 ± 1 °C, with a 16h/8h photoperiod. Manual agitation was performed once or twice a day. The cultivation was carried out in ASM-1 inorganic medium, with inoculations performed every 2 weeks. All materials used for the inoculation process were subjected to autoclaving at 15 min and 121 °C. A laminar flow hood was sterilized with 70% alcohol and ultraviolet light for 15 min before each inoculation procedure. The culture used in this study was in its growth phase.

### 2.3 Collection and Preparation of the Water Sample

The study water was composed of natural water with a concentrated volume of cyanobacterial cells added. The water was sourced from a water reservoir in Curitiba, Paraná. After collection, the water was filtered through a 20 μm plankton net to remove impurities from the natural source. Experiments were conducted immediately after the collection.

The cell concentrate was prepared by centrifuging the pure cyanobacterial culture at 4000 rpm for 10 min using a Daiki DT4500 centrifuge. The process was conducted in Falcon tubes containing 40 mL of culture. Following centrifugation, the supernatant was discarded into 5 L containers containing 5% chlorine for disinfection, given that it was not used in the experiments, and to avoid environmental contamination. The cell concentrate, obtained after centrifugation, was resuspended in spring water (i.e., untreated water collected from a local source) to achieve the target cell density. This approach was employed to simulate natural cyanobacterial blooms and minimize interference from extracellular metabolites in the laboratory culture. Subsequently, to prepare the cell concentrate, cell density was determined by counting cells using an optical microscope and a Neubauer or Sedgewick-Rafter chamber. The initial conditions were as follows: cell density of 5.0 × 10^5^ cells mL^–1^, turbidity = 40 NTU, pH = 8.0, and temperature = 20 ± 1 °C.

### 2.4 MO and PAC Coagulant

The natural coagulant was prepared by using *Moringa oleifera* seeds. MO seeds were crushed and homogenized by sieving (270 mesh). A solution containing 10 g L^–1^ seeds was dissolved in a saline solution of CaCl_2_ and stirred for 30 min at 400 rpm. After this step, the solution was filtered using a qualitative filter and then a fiberglass filter (0.45 μm). The different concentrations were obtained from a stock solution prepared with seed powder at a concentration of 10 g L^–1^.

To perform the PAC tests, a coagulant solution was initially prepared at a concentration of 10 g L^–1^. The required volumes for the different dosages were then measured in this solution.

### 2.5 Dissolved Air Flotation (DAF)

The C/F/DAF experiments were conducted using the jar test (M. 218 LDBF) equipment from Ethik Technology (Figure 1). This equipment includes a pressurization chamber and transparent acrylic jars for coagulation/flocculation/flotation. The base of each jar is composed of two acrylic plates separated by 5 cm. These plates are perforated with 121 holes to create adequate head loss, ensuring uniform distribution of the saturated water. This saturated water is provided by a saturation chamber connected to the system. An air compressor supplies the air for water saturation in the chamber.

**Figure 1:**
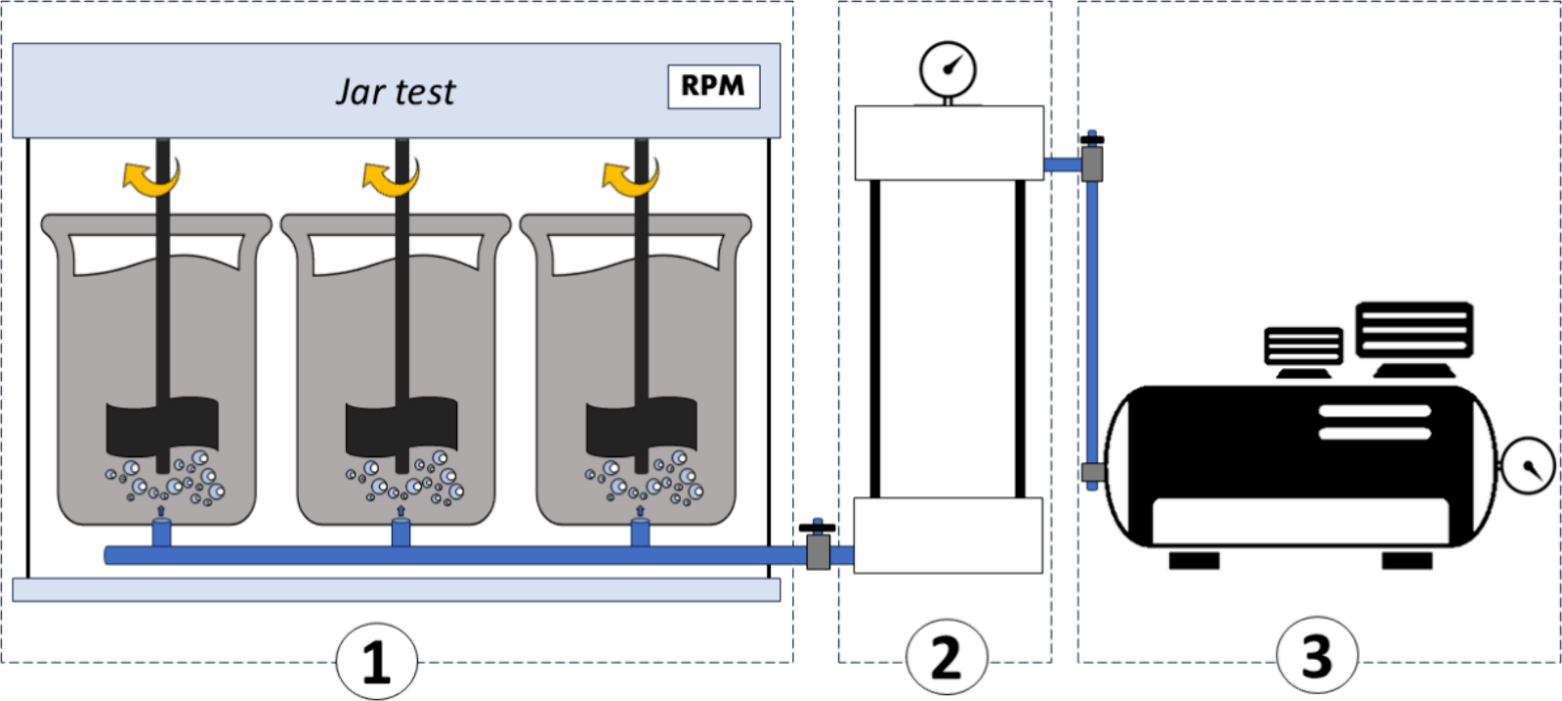
Dissolved air flotation (DAF) equipment used for conducting the tests: (1) jar test, (2) water saturation chamber, and (3) air compressor.

The operational parameters were a rapid mixing gradient of 1000 s^–1^ for 10 s, a slow mixing gradient of 15 s^–1^ for 15 min, a saturation pressure of 400 kPa for 8 min, a recirculation rate of 10%, and a flotation velocity of 5 cm m^–1^ (2 m^3^ m^–2^ day).

### 2.6 Study of Cyanobacteria Removal by C/F/DAF

The experimental development was carried out in three main stages, focusing on the efficiency of removing three parameters: cell density, turbidity, and color.

#### 2.6.1 Determination of the Ideal Dosage

The species CR and MA with a cell density of 5.0 × 10^5^ cells mL^–1^ were used to determine the optimal dosage of MO. The tested dosages were 5, 10, 20, 40, and 80 mg L^–1^.

#### 2.6.2 Evaluation of Coagulant Proportions

Based on the ideal dosage identified, different proportions of coagulants (MO and PAC) were evaluated in five distinct combinations: 100%MO:0%PAC, 75%MO:25%PAC, 50%MO:50%PAC, 25% MO:75%, and 0%MO:100% PAC.

#### 2.6.3 Influence of Seasonal Variation

The impact of seasonal variation on cyanobacteria populations was assessed, considering reservoir water under natural conditions (without cyanobacteria blooms). Different cellular proportions of the studied species were simulated: 100%MA:0%CR, 75%MA:25%CR, 50%MA:50%CR, 25%MA:75%CR, and 0%MA:100%CR.

### 2.7 Water Characterization

The characterization analyses were conducted with residual aluminum (mg L^–^^1^) using the colorimetric ECR method (Thermo Hiper analyzer), electrical conductivity (μS cm^–1^) with a digital conductivity meter, and turbidity (NTU) using the nephelometric method and a turbidimeter.

The total dissolved carbon (TDC) and dissolved inorganic carbon (DIC) measurements were performed using a Thermo HiperTOC analyzer (combustion method at 680 °C and CO_2_ detection), and the concentration of dissolved organic carbon (DOC, mg L^–^^1^) was determined by the difference between the TDC and DIC concentrations in water. The samples were filtered using a cellulose acetate membrane (0.45 μm), stored in carbon-free glass bottles, preserved with hydrochloric acid (pH <2.0), and kept under refrigeration.

The organic matter was characterized by using specific ultraviolet absorption (SUVA) measurement. This measurement was based on the absorbance of the sample at 254 nm (eq 1):

$$\mathrm{SUVA}=\frac{{\mathrm{UV}}_{254\mathrm{nm}}\times 100}{\mathrm{DOC}}$$ 1

where SUVA is the specific absorbance (cm^–1^ mg L^–1^); DOC is the dissolved organic carbon (mg L^–1^); UV_254 nm_ is the absorbance of the sample at 254 nm.

#### 2.7.1 Color Analysis

The color analysis was performed by using the photoelectric method. A standard solution at 500 HU (Hazen unit) was prepared by dissolving 1.000 g of CoCl_2_·6H_2_O and 1.245 g of potassium chloroplatinate in 1000 mL of hydrochloric acid 100 mL (10%). A standard curve was constructed at 455 nm with a UV/vis spectrophotometer (Global Trade Technology). The standard curve can be viewed in the Supporting Information (Figure S1).

### 2.8 Fluorescence Spectrum

The fluorescence analyses were performed by using a Cary Eclipse spectrofluorometer (Varian, Inc.), equipped with a quartz cuvette with an optical path length of 1 cm. Scanning was carried out from 200 to 750 nm. Ultrapure water was used as an analytical control. Inert glass jars were used for sample collection, which were cleaned with nitric acid and then calcined in a muffle furnace at 500 °C for 5 h. After collection, the samples were filtered through a 0.45 μm cellulose acetate membrane and then acidified with 1 mol L^–1^ HCl (pH = 3.0).

## 3 Results and Discussion

### 3.1 Influence of Natural Coagulant Dosage

Table S1 shows the characterization of the water used in this stage of the study. The color of the water varied due to the amount of natural pigment produced by each cyanobacteria species. Figure 2 shows the influence of the natural coagulant dosage on the turbidity of water containing two cyanobacteria species. The dosage of 20 mg L^–1^ resulted in the highest turbidity removal efficiency, with 81% for MA, whereas for CR, it was 82%. However, according to the Tukey test (*p* ≤ 0.05), there is no significant difference in turbidity removal for the MA samples between the 20, 40, and 80 mg L^–1^ dosages. Camacho et al.^24^ evaluated different dosages of a natural coagulant for turbidity removal in water containing cyanobacteria, using MO seeds extracted with ethanol, pressurization, and moringa powder. The authors determined that the most effective dosage for highly turbid water was 50 mg L^–1^ in the coagulation/flocculation/sedimentation process. Gandiwa et al.^25^ also found 50 mg L^–1^ to be the optimal concentration for turbidity removal through the coagulation process when MO was used in raw water treatment. The authors observed that increasing the dosage of the natural coagulant beyond the optimal concentration increased turbidity. This occurs due to the coagulant’s high concentration and the particles’ restabilization.^25,26^

**Figure 2:**
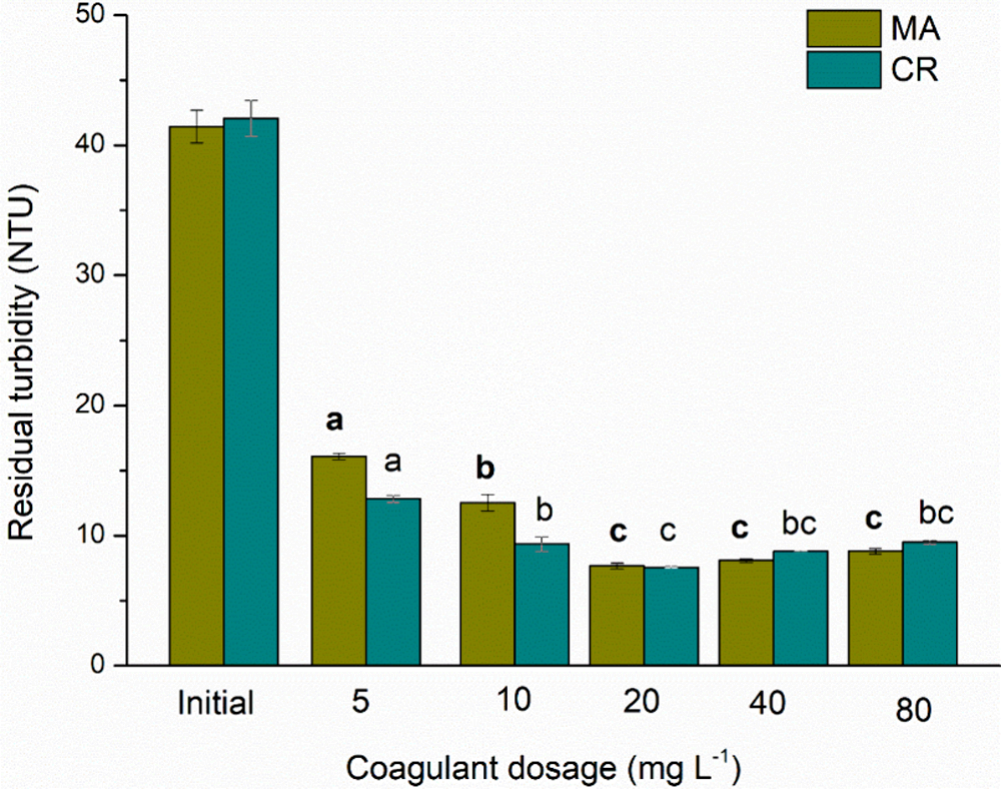
Residual turbidity for C/F/DAF treatment with different concentrations of MO for [MA] = 5.73 × 10^5^ cells mL^–1^ and [CR] = 5.47 × 10^5^ cells mL^–1^ species.

Figure 3 shows the color residuals for each evaluated cyanobacterial species. The dosage of 20 mg L^–1^ resulted in the lowest residual, with removal percentages of 67% for MA and 76% for CR. However, there was no significant difference (*p* ≤ 0.05) in color removal within the 10, 20, 40, and 80 mg L^–1^ dosages for species MA. In the case of species CR, there was no significant difference (*p* ≤ 0.05) within the dosages of 10, 20, and 40 mg L^–1^. Kenea et al.^27^ investigated the removal of various parameters using aloe vera plants and MO seeds as natural coagulants. The authors achieved a color removal of 87.1% with application of the coagulant mixture in surface waters. In this study, the color from natural pigments produced by cyanobacteria may have influenced the color removal efficiency.

**Figure 3:**
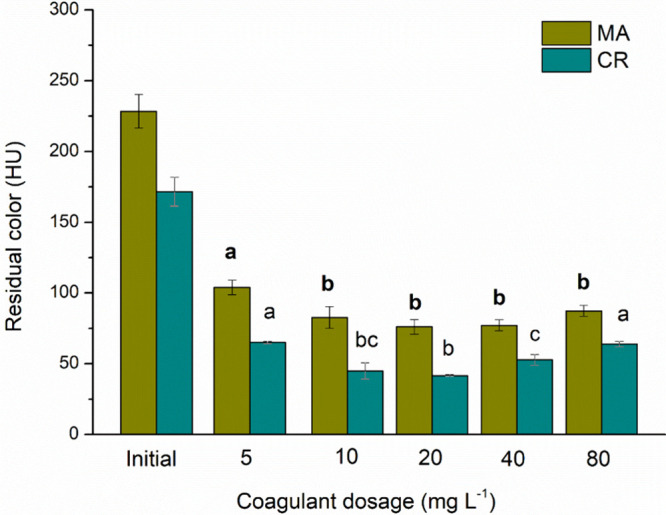
Residual color for C/F/DAF treatment with different concentrations of MO for [MA] = 5.73 × 10^5^ cells mL^–1^ and [CR] = 5.47 × 10^5^ cells mL^–1^ species.

Figure 4 shows the effect of the natural coagulant dosage on cell density after treatment. The MA species’ cell removal efficiency was 88% with a dosage of 20 mg L^–1^ of MO. However, there was no significant difference (*p* ≤ 0.05) between the 20 and 40 mg L^–1^ dosages. For the CR species, a dosage of 20 mg L^–1^ achieved 97% cell removal efficiency. Nevertheless, there was no significant difference (*p* ≤ 0.05) among the 10, 40, and 80 mg L^–1^ dosages. Teixeira et al.^28^ removed approximately 80% of MA cells in the C/F/DAF system. On the other hand, Carvalho et al.^29^ removed 78.8% of MA. In both studies, MO was used as a natural coagulant at a dosage of 50 mg L^–1^.

**Figure 4:**
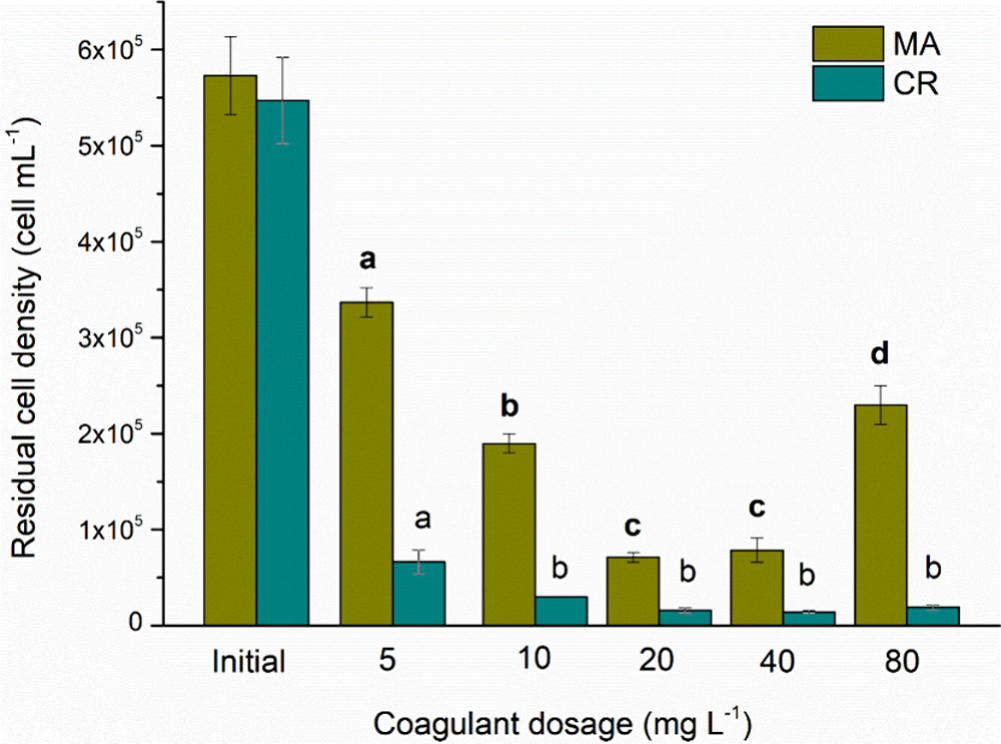
Residual cell density for C/F/DAF treatment with different concentrations of MO for [MA]_0_ = 5.73 × 10^5^ cells mL^–1^ and [CR]_0_ = 5.47 × 10^5^ cells mL^–1^ species.

During the tests, the addition of the natural coagulant did not affect the pH of the water, with a maximum reduction of 0.05%, consistent with previous studies.^30,31^ Camacho et al.^24^ emphasize that using MO as a natural coagulant represents an advantage. In contrast, aluminum sulfate requires pH adjustment to enhance coagulation efficiency and subsequently raises process costs. MO does not require such adjustments, making the process more cost-effective.

Figure 5 shows principal component analysis (PCA). PCA was applied to reduce the dimensionality of the data, identify patterns, and facilitate the interpretation of the results. For the studied cyanobacteria species, PC1 and PC2 explained 95% of the variance for MA and 98% for CR, providing discriminatory information about the samples. The results showed that the samples with 40 and 80 mg L^–1^ of the natural coagulant MO clustered together for both microorganisms with higher Abs254 and conductivity readings. In contrast, the sample with 5 mg L^–1^ maintained parameters similar to those of the initial water. The dosage of 20 mg L^–1^ showed reduced color and turbidity parameters for both species, with minimal impact on Abs254 and conductivity. Therefore, 20 mg L^–1^ was selected for the next stage of the study.

**Figure 5:**
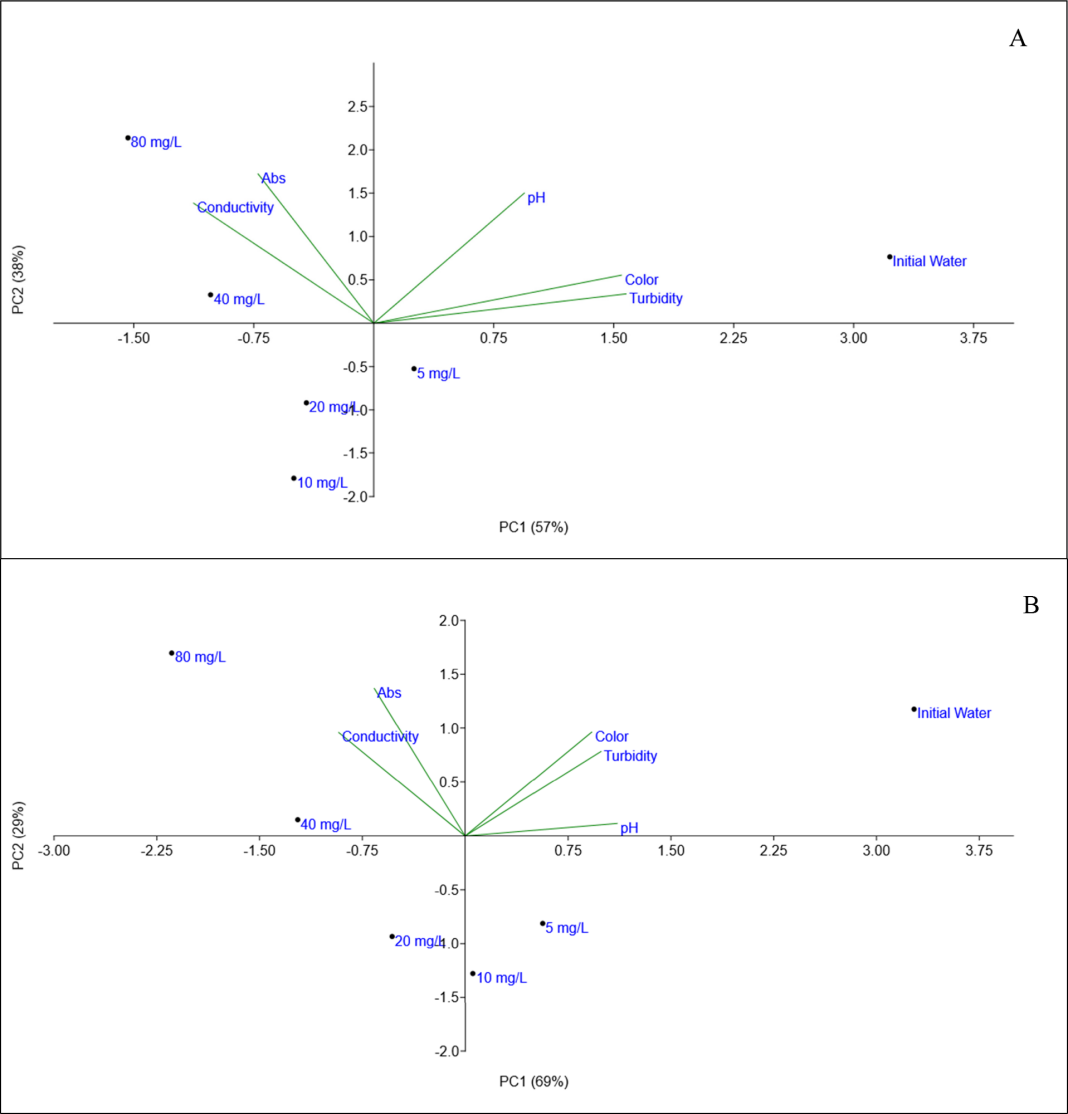
PCA of water quality parameters in the analysis of the optimal dosage of MO for *Microcystis aeruginosai*­(A) and *Cylindrospermopsis raciborskii* (B).

### 3.2 Influence of Different Proportions of Coagulants MO and PAC on the Removal of Cyanobacteria, Evaluated Individually

The influence of coagulant ratios was analyzed with regard to color, turbidity, and cell density. The coagulants used were MO and PAC. PAC was used as the metallic coagulant because it is one of the most widely used chemical coagulants in water treatment plants. Additionally, compared to aluminum sulfate, the hydrolysis of the aluminum cation is slower, which facilitates the interaction of the coagulant’s charges with the particles present in the water.^32^ Therefore, the protein network formed by MO is expected to interact with PAC, released aluminum, and the contaminants.

Table S2 shows the characterization of the water used in the study of different proportions of the MO and PAC. Figures 6 and 7 show the influence of coagulant proportions on residual turbidity and color, respectively. As the proportion of PAC increases, the residual color and turbidity decrease for both cyanobacteria species. The 0% MO and 100% PAC ratios were the most effective for these parameters. It also resulted in higher residual soluble aluminum levels, as shown in Figure 8. Soluble aluminum in water can present serious public health risks,^33,34^ and insoluble aluminum accumulating in the sludge generated during treatment can make the sludge unsuitable for use as a resource. However, the combination of PAC with the MO saline extract shows satisfactory removal of color and turbidity. This coagulant combination has the benefit of reducing soluble aluminum in the treated water, as well as particulate aluminum in the sludge, making it more biodegradable and potentially suitable for applications beyond landfill cover.^35^ Regarding pH, no significant changes were observed in the pH of the treated water, regardless of the coagulant proportions, with a maximum reduction in pH of 0.07%.

**Figure 6:**
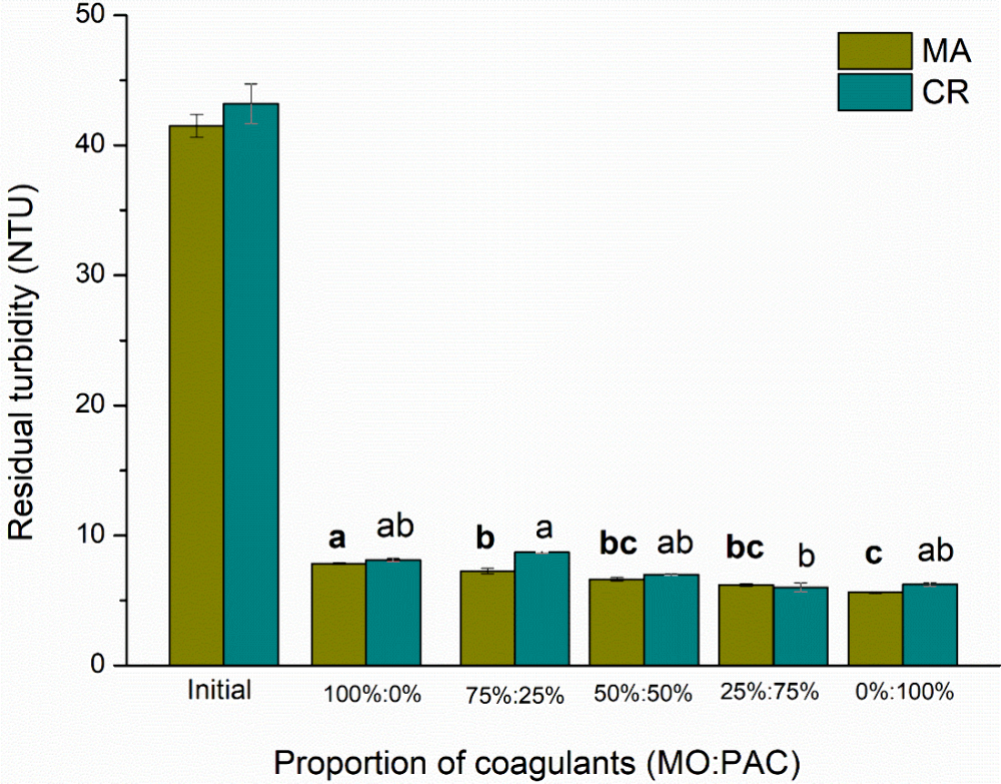
Residual turbidity for C/F/DAF treatment with different coagulant ratios for [MA] = 6.60 × 10^5^ cells mL^–1^ and [CR] = 5.68 × 10^5^ cells mL^–1^ species.

**Figure 7:**
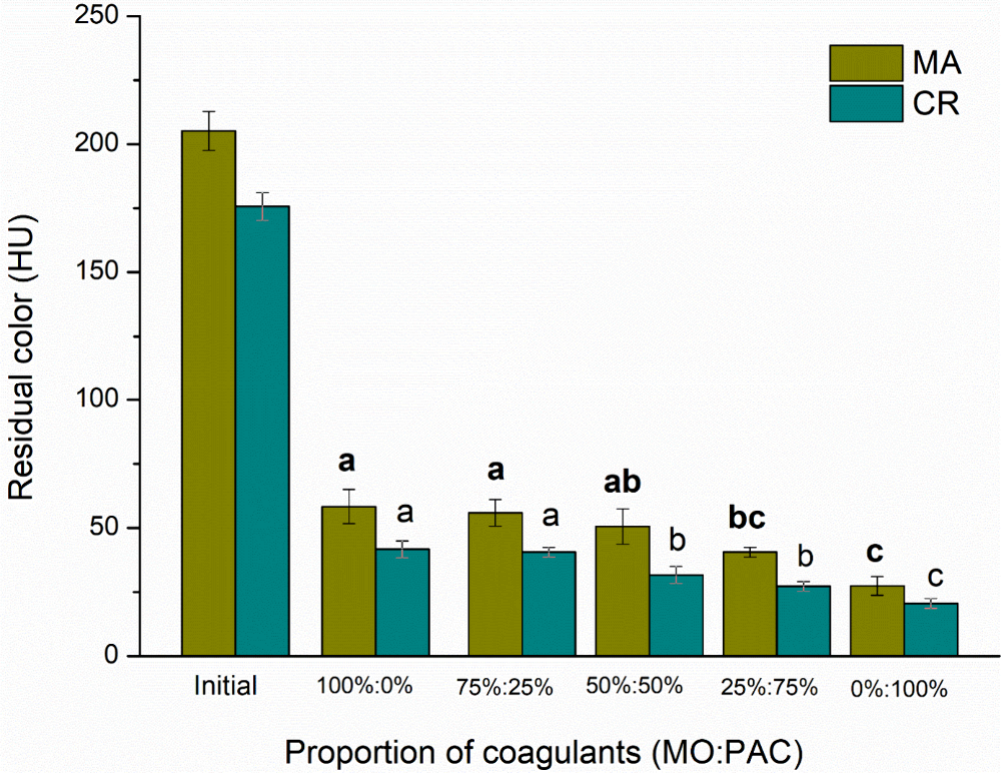
Residual color for C/F/DAF treatment with different coagulant ratios for [MA] = 6.60 × 10^5^ cells mL^–1^ and [CR] = 5.68 × 10^5^ cells mL^–1^ species.

**Figure 8:**
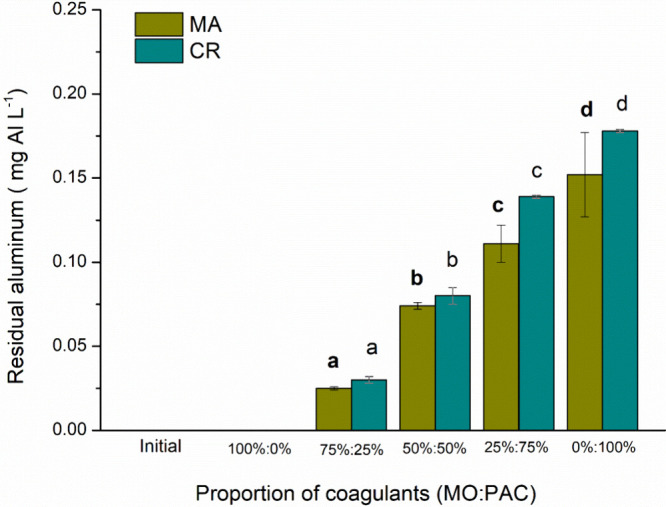
Residual soluble aluminum (mg Al L^–1^) for the proportions of MO and PAC for [MA] = 6.60 × 10^5^ cells mL^–1^, [CR] = 5.68 × 10^5^ cells mL^–1^ species, and [Initial] = untreated water.

Figure 9 shows the impact of various coagulant proportions on the residual cell density of the cyanobacteria. The 75% MO and 25% PAC dosage achieved approximately 95% removal of cell density for both cyanobacteria species, resulting in the lowest residual aluminum levels (Figure 8). This effectiveness can be attributed to the role of coagulants in the dissolved air flotation (DAF) process. Cyanobacteria cells, such as *Microcystis* sp., with diameters of 3–7 μm, have difficulty colliding with air bubbles due to their small size. The coagulant increases particle size and destabilizes them, enhancing the process’s efficiency.^36^

**Figure 9:**
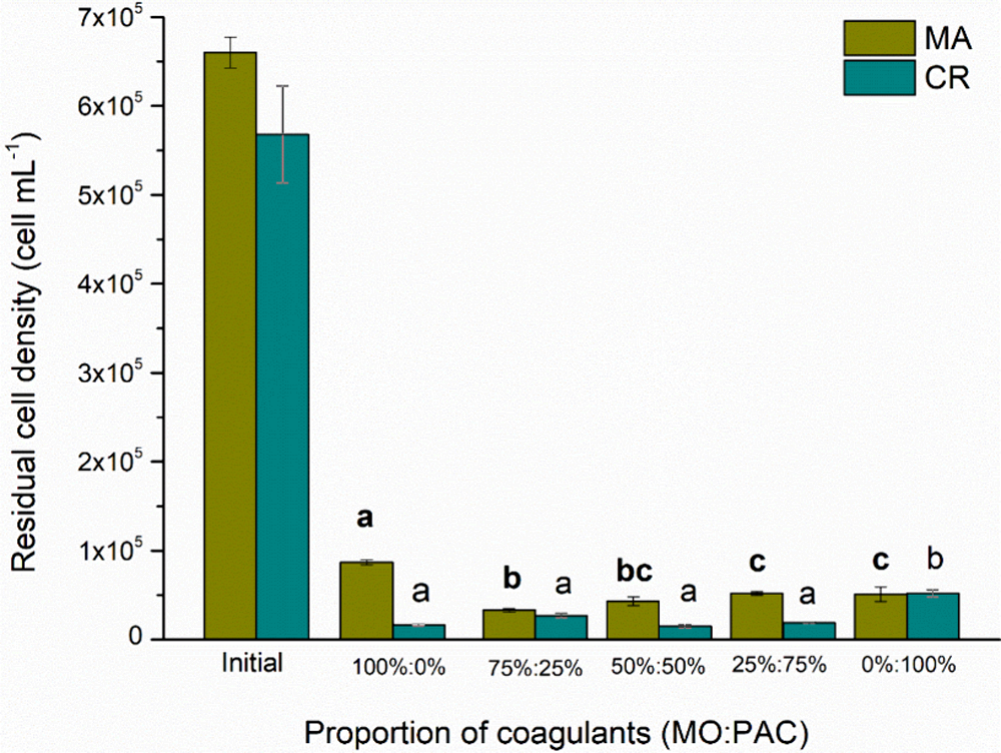
Residual cell density for C/F/DAF treatment with different coagulant ratios for [MA]_0_ = 6.60 × 10^5^ cells mL^–1^ and [CR]_0_ = 5.68 × 10^5^ cells mL^–1^ species.

The improvement in the C/F/DAF process efficiency when the combination of MO and PAC was used can be attributed to the synergistic action between the two coagulants. The proteins present in MO seeds have cationic polyelectrolyte characteristics^12,13^ and are positively charged. They help to attract and aggregate organic and cellular matter, such as cyanobacterial cells, in the medium. This interaction is enhanced by the presence of aluminum chloride, which, when acting in conjunction with MO, promotes greater agglomeration and flocculation of the particles, resulting in improved efficiency in the removal of impurities such as turbidity, color, and cells. Therefore, the MO-PAC combination proves to be more effective than the use of each coagulant individually, providing a more efficient water treatment.

Figure 10 shows the PCA analysis for the different proportions of the coagulants used. For the studied cyanobacteria species, PC1 and PC2 explained 86% of the variance for MA and 98% for CR, providing discriminatory information about the samples. The analysis shows that samples with higher proportions of PAC tend to cluster at 25% MO:75% PAC and 0% MO:100% PAC. Similarly, the samples with a higher proportion of MO also cluster together. Additionally, the electrical conductivity parameter is higher for the samples with greater proportions of the natural coagulant. This can be attributed to the saline extract of CaCl_2_ used to prepare the natural coagulant. A previous study^37^ observed that treating wastewater with oil-free MO resulted in an increase of up to 8% in the conductivity of the treated water at the highest coagulant dosage used (30 mg L^–1^). This increase was attributed to the introduction of salts into the water from the pretreatment of the seeds used in the process.

**Figure 10:**
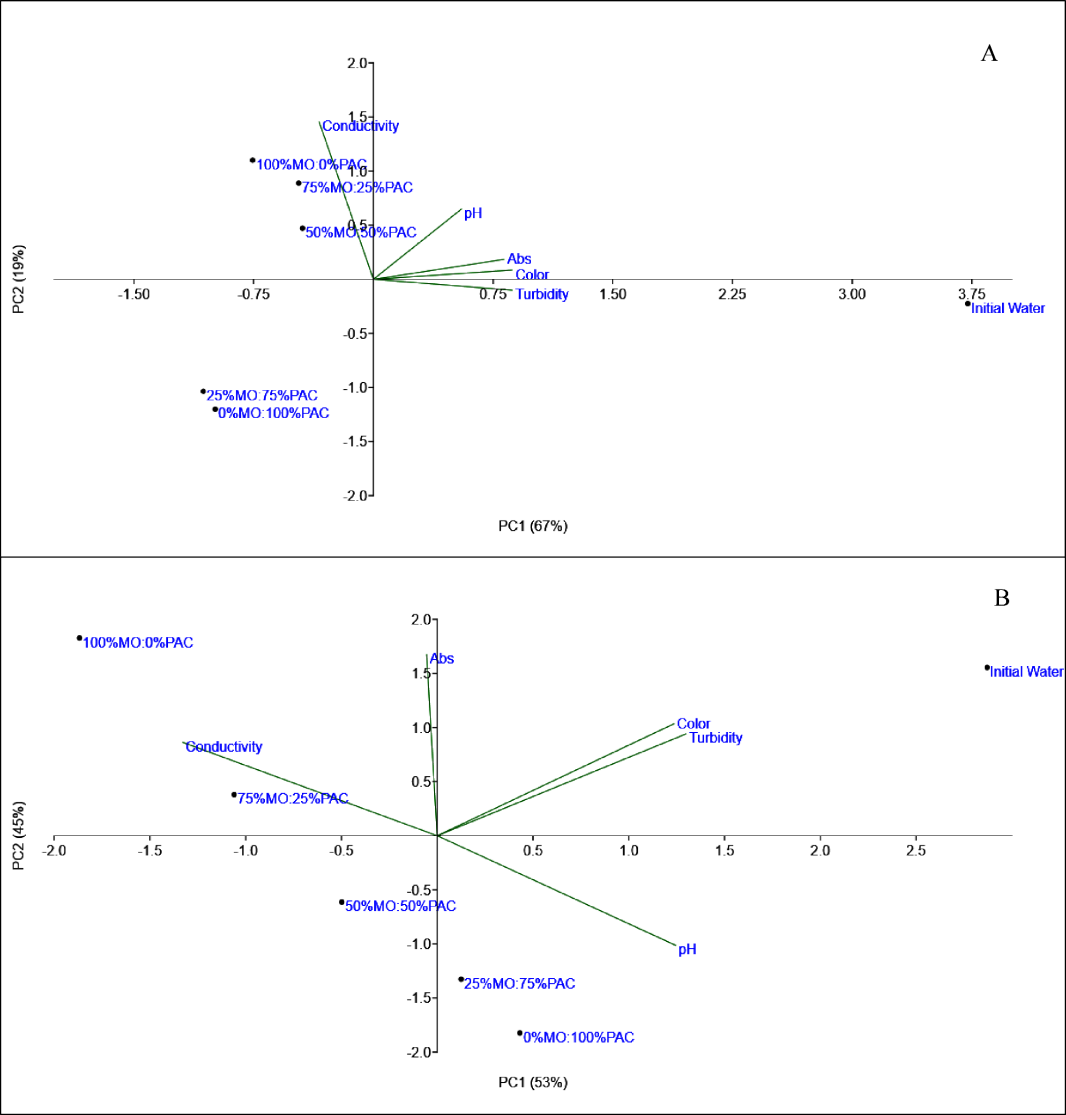
PCA analysis of water quality parameters in the study of the influence of different proportions of PAC and MO for *Microcystis aeruginosai*­(A) and *Cylindrospermopsis raciborskii* (B) species.

Comparing the PCA results reveals a clear distinction between the samples along the principal components, indicating a good initial separation of the variables (Figure 5). With the addition of different coagulant proportions, the samples start to display more defined clustering patterns (Figure 10), suggesting that the coagulant proportion directly influences the behavior of the variables. A shift in the principal axes with the introduction of PAC proportions is also evident, indicating that the samples respond differently as the coagulant concentration increases. This can be attributed to how variance is redistributed among the components: as the coagulant is added in varying proportions, some variables contribute more to the principal components, altering the explained variance values. Additionally, the presentation of the variables also changes with increasing proportions. Some variables become more correlated as the coagulant concentration rises, suggesting a more direct effect on these specific responses, while others remain stable. These changes in variance, variable distribution, and sample arrangement indicate a significant interaction between coagulants and microorganisms, suggesting that coagulant proportions impact the effectiveness of the C/F/DAF treatment.

For the next stage of this study, the 75% MO:25% PAC proportion was selected due to its high percentage of cell density removal and lower residual aluminum generation.

### 3.3 Evaluation of the Influence of Seasonal Variation of Cyanobacteria Populations on the Efficiency of Associated Coagulants

Table S3 shows the characterization of the water used in this study stage. The color of the water is derived from humic and fulvic substances, products of the decomposition of sediments and plant matter.^38^ However, the addition of cyanobacteria increases this parameter due to the pigment produced by these microorganisms called phycocyanin. A previous study^39^ shows that the level of dissolved organic carbon (DOC) in natural river water worldwide generally ranges from 2 to 10 mg L^–1^, which is consistent with the values found in this study. Using the DOC and Abs_254 nm_ values, the SUVA254 nm value was calculated. According to the values obtained, the water exhibits characteristics of natural organic matter associated with hydrophobic bases or neutral compounds.^40^

At this stage, the cell density was adjusted to reflect seasonal changes in cyanobacteria populations. Table S4 shows the different proportions studied and their corresponding cell densities. Table S5 shows the efficiency of color and turbidity removal with different proportions of cyanobacteria. A decrease in the color removal efficiency was observed in the presence of cyanobacteria. Specifically, the efficiency of color removal was 84% in cyanobacteria-free water, whereas the mean value with cyanobacteria was approximately 74.6%. This decrease can be explained by the presence of cyanobacteria in the water. These microorganisms have a negative charge, similar to that of some natural organic matter. As a result, they compete for the coagulant’s active sites, reducing the coagulation process’s efficiency.^41^

Figure 11 shows the residual color and turbidity among the various cell proportions tested. No significant differences in color removal were observed among the various cell proportions tested, with a mean removal value of 74.6%. However, the highest turbidity removal efficiency was achieved with a 50% proportion of MA and 50% of CR, resulting in a 94% turbidity removal rate (Table S5). In comparison, a previous study^42^ achieved 69% color removal and 60% turbidity removal using a coagulation/flocculation/DAF process with 40 mg L^–1^ aluminum sulfate as the coagulant. In this study, the removal efficiencies for water free of cyanobacteria were 84% for color and 88% for turbidity.

**Figure 11:**
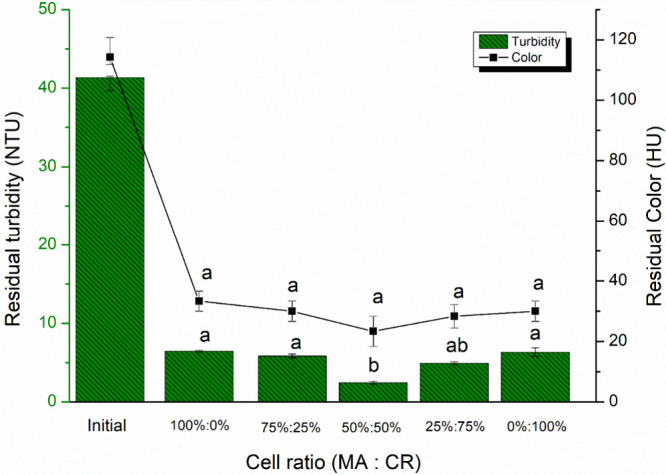
Residual color (HU) and turbidity (NTU) for the 75% MO:25% PAC proportion for the different cellular proportions of MA and CR species.

Figure 12 shows the residual cell density for the different cell proportions studied. Although no significant differences (*p* ≤ 0.05) in cell removal efficiencies were observed among the various proportions, the 50% MA and 50% CR ratio achieved ≈99% cell removal. According to Henderson et al.,^43^ cell removal efficiency is more closely correlated with the electric charge of the material or microorganism than with cell morphology itself. In addition, the samples did not undergo filtration, resulting in residuals of 4.32 × 10^3^ cells mL^–1^ for MA and 7.35 × 10^3^ cells mL^–1^ for CR. If filtration had been applied after the C/F/DAF process, then the removal of cyanobacteria cells would probably have been higher, potentially even complete.

**Figure 12:**
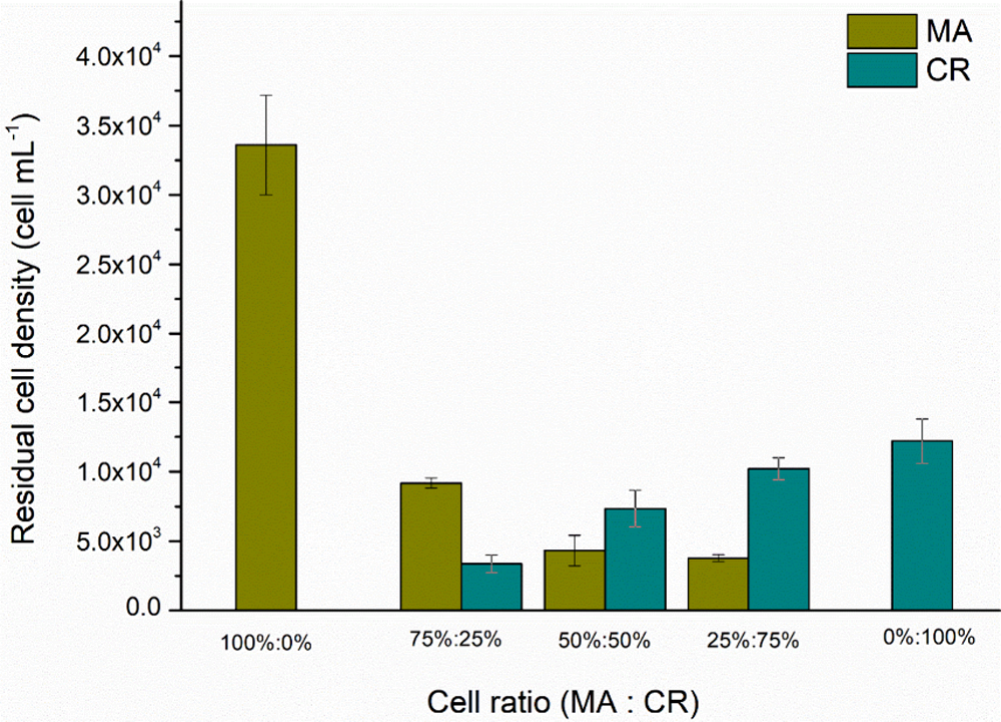
Residual cell density for the 75% MO:25% PAC proportion for the different cellular proportions of MA and CR species.

### 3.4 Organic Matter Analysis

Table 1 shows the TDC and DOC values of the analyzed samples. The cell integrity of the microorganisms was assessed by analyzing changes in DOC throughout the processes. It was observed that the DOC did not show significant variations across the different cell densities, indicating that cell integrity was preserved in most cases. However, at the dosage of 0% MA and 100% CR, a detectable increase in dissolved organic matter in the medium was observed, suggesting that only this microorganism may have suffered cell damage. Therefore, the other cyanobacteria proportions did not compromise cell integrity, maintaining DOC stability. In a previous study,^36^ the removal of cyanobacteria from water using the C/F/DAF process efficiently eliminated *M. aeruginosa* without releasing toxins into the water, supporting the findings of this study regarding the preservation of cell integrity under certain conditions. Additionally, the analysis of TDC showed slight variations across different treatments, indicating a minimal impact on TDC levels in most cases.

**Table 1:** Dissolved Organic Carbon (DOC) and Total Dissolved Carbon (TDC) (mg L^–1^) under Different C/F/DAF Treatment Conditions Using a Coagulant Ratio of 75% MO:25% PAC^a^

	initial water	initial water (after C/F/DAF)	100%:0% (MA:CR)	75%:25% (MA:CR)	50%:50% (MA:CR)	25%:75% (MA:CR)	0%:100% (MA:CR)
DOC (mg L^–1^)	5.35 ± 0.60	6.58 ± 0.04	6.66 ± 1.10	6.42 ± 0.72	6.22 ± 1.30	6.89 ± 0.71	9.50 ± 0.01
TDC (mg L^–1^)	7.34 ± 0.00	8.66 ± 0.00	8.69 ± 1.10	8.50 ± 0.64	8.40 ± 1.35	8.99 ± 0.82	11.42 ± 0.08

a Initial water = water without cyanobacteria.

### 3.5 Fluorescence Spectra

Figure 13 shows the fluorescence spectra used to examine changes in the fluorescence of organic matter in the study water. It includes data for the water treated with C/F/DAF (50% MA and 50% CR) and without cyanobacteria. Applying a proportion of 75% MO and 25% PAC, the C/F/DAF treatment efficiently removed proteins, fulvic acids, and humic acids in the water for all samples. Additionally, the area corresponding to the excitation and emission wavelengths between 600 and 700 nm can be attributed to the presence of cyanobacteria. This is evident because the same areas of emission and excitation did not show these peaks in the water without cyanobacteria. According to previous studies,^44,45^ cyanobacteria pigments are excited at higher wavelengths, with an excitation maximum between 550 and 680 nm and an emission range of 640–680 nm.

**Figure 13:**
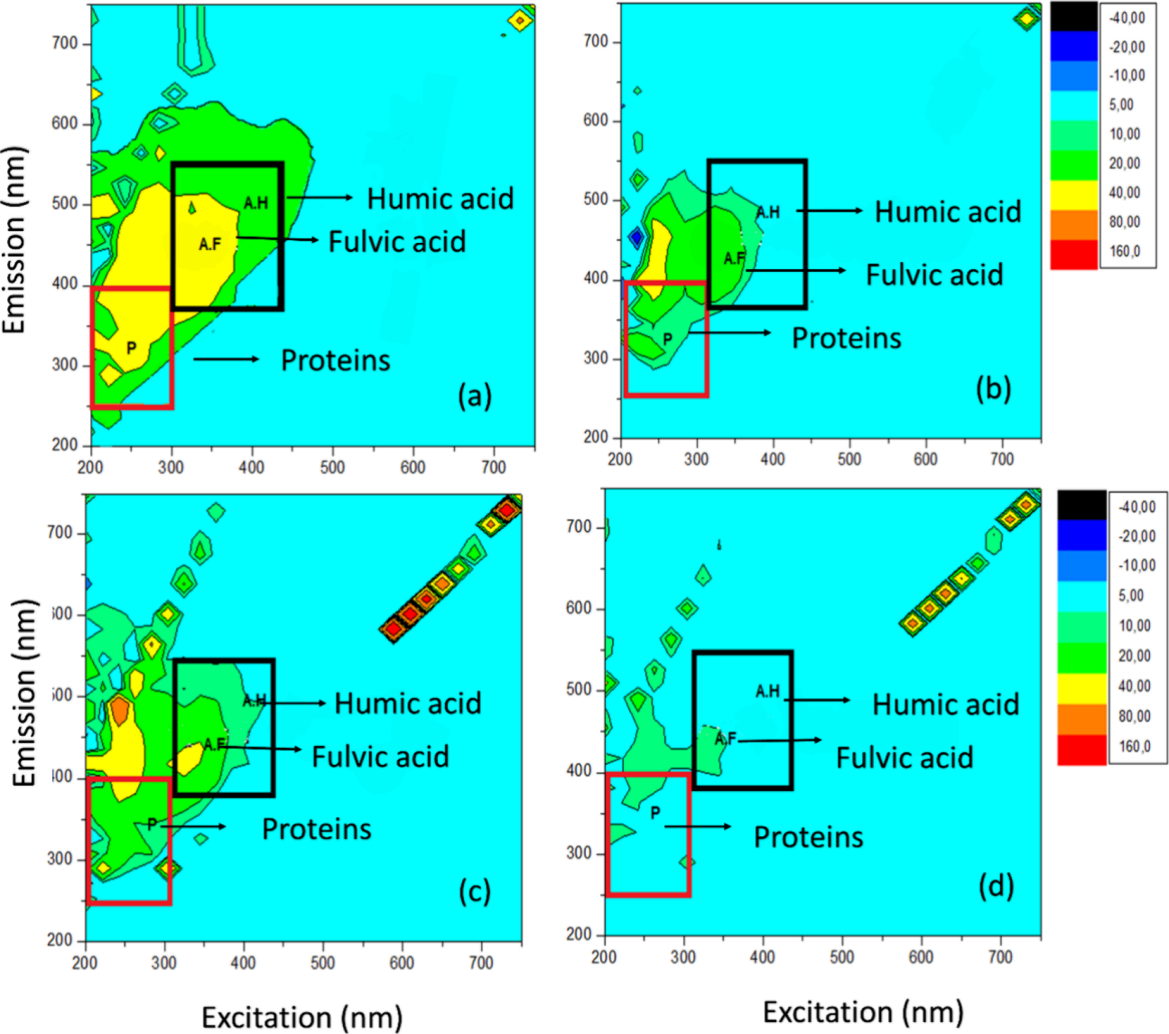
Fluorescence spectra for (a) cyanobacteria-free water before treatment, (b) cyanobacteria-free water after treatment, (c) water 50% MA:50% CR before treatment, and (d) water 50% MA:50% CR after treatment.

Figure 14 shows the synchronous fluorescence spectroscopy for the study of water. The peak in the excitation wavelength range of 275–300 nm indicates that the source water already contained an initial concentration of proteins, regardless of the presence of cyanobacteria. After the treatment, a significant increase in this excitation range was observed, which can be attributed to the proteins from the natural coagulant. After the treatment, the peak related to proteins increased. This can be attributed to the protein fractions in the natural coagulant. A previous study^46^ identified that 97% of the proteins in MO seeds are globulins and albumins with high coagulation power. Additionally, the synchronous fluorescence graph shows that the wavelength range of 700–750 nm indicates the presence of cyanobacteria in the water. This range exhibited peaks only in the samples containing cyanobacteria, while the study water without cyanobacteria did not show significant peaks in this region.

**Figure 14:**
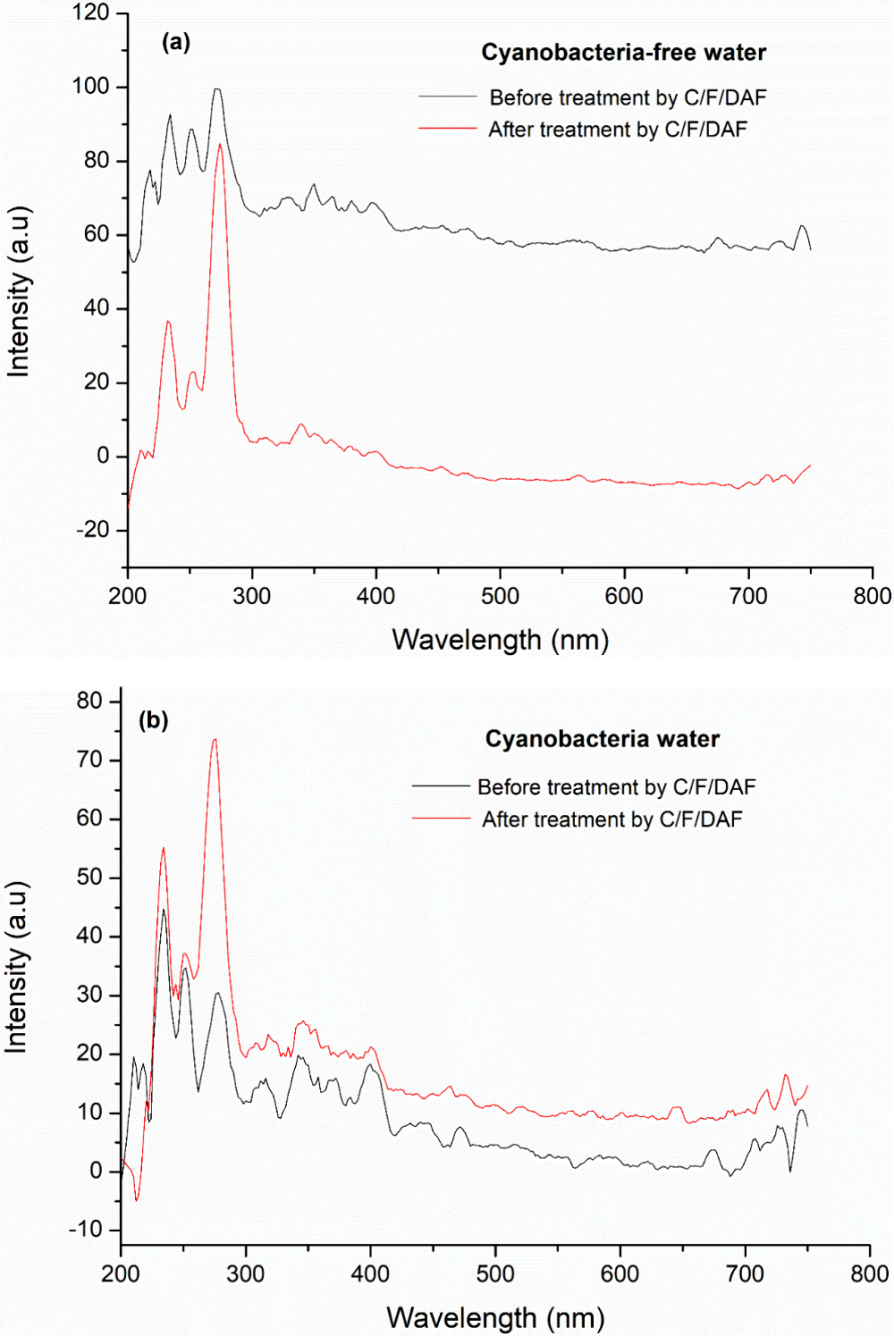
Synchrotron fluorescence spectra of (a) the initial study water and (b) the water treatment using a coagulant ratio of 75% MO:25% PAC.

## 4 Conclusions

The combination of MO and PAC coagulants was effective for the removal of cyanobacteria by C/F/DAF. The saline extract of MO demonstrated high efficacy as a coagulant in removing turbidity, color, and cell density, with an optimal dosage of 20 mg L^–1^. The combination of coagulants with 75% MO and 25% PAC proved to be the most effective, indicating that this mixture is suitable for treating cyanobacteria in C/F/DAF processes while achieving a lower residual aluminum concentration (0.03 mg L^–1^). Cell integrity was observed in most cases of C/F/DAF treatment. Furthermore, the fluorescence spectrum revealed that proteins, fulvic acids, and humic acids present in water were also removed after treatment. Seasonal variation showed that cyanobacteria species’ morphology and cell density did not affect the C/F/DAF treatment. This study offers valuable insights into the removal of cyanobacteria from water sources and contributes to enhancing dissolved air flotation processes. It highlights the effectiveness of the coagulant combination and the reduction of residual aluminum impact, which could lead to a more sustainable and efficient treatment, minimizing potential risks associated with conventional chemical coagulants, and improving water quality.

## Supplementary Material



## Notes

Characterization of water used at each experimental stage and standard curve for color quantification (PDF)
