# Supplementary material for: Moringa oleifera and Polyaluminum Chloride: Coagulant Combinations for Cyanobacteria Removal in Drinking Water
Source: ACS Omega. 2025 Aug 22;10(35):39712–23. doi: 10.1021/acsomega.5c03206 (PMC12423882; doi:10.1021/acsomega.5c03206)

Moringa oleifera and Polyaluminum Chloride: Coagulant Combinations for Cyanobacteria Removal in Drinking Water

Camila Laschiwitz Beghetto[1], Rúbia Martins Bernardes Ramos[1], Pablo Inocêncio Monteiro[2], Fatima de Jesus Bassetti[1], Lucila Adriani de Almeida Coral[1]*

Posgraduate Program in Environmental Science and Technology, Federal University of Technology – Paraná (UTFPR), R. Deputado Heitor Alencar Furtado, 5000 – CIC, CEP 81280-340 Curitiba, PR, Brazil [1]

Posgraduate Program in Food Engineering, Chemical Engineering Department, Federal University of Parana, 81531-980, Curitiba, PR, Brazil [2]

* Corresponding author. Email: lucilacoral@utfpr.edu.br

Supplementary Material

Table S1. Characterization of the study water used to determine the optimal dosage of MO for the cyanobacteria species *M. aeruginosa* and *C. raciborskii*

Parameters	*M. aeruginosa (MA)*	*C. raciborskii (CR)*
Turbidity (NTU)	41.43 ± 1.28	42.08 ± 1.38
pH	8.09 ± 0.04	7.78 ± 0.08
Color (HU)	228.33 ± 11.78	171.66 ± 10.27
Abs 254 nm (cm^{-1})	0.049 ± 0.005	0.052 ± 0.003
Cell density (cell mL^{-1})	$5.73 \times 10^{5} \pm 4.04 \times 10^{4}$	$5.47 \times 10^{5} \pm 4.5 \times 10^{4}$
Conductivity (µS cm^{-1})	150.0 ± 0.050	120.0 ± 0.050

Table S2. Characterization of the study water used in tests with different proportions of MO saline extract and PAC coagulants for the MA and CR cyanobacteria species

Parameters	*M. aeruginosa* (MA)	*C. raciborskii* (CR)
Turbidity (NTU)	41.50 ± 0.87	43.20 ± 1.53
pH	7.83 ± 0.13	7.62 ± 0.06
Color (HU)	205.20 ± 7.58	175.66 ± 5.47
Abs 254 nm (cm^{-1})	0.056 ± 0.02	0.049 ± 0.02
Cell density (cell mL^{-1})	$6.60 \times 10^{5} \pm 1.73 \times 10^{4}$	$5.68 \times 10^{5} \pm 5.46 \times 10^{4}$
Conductivity (µS cm^{-1})	130.00 ± 0.010	140.00 ± 0.0050
Residual aluminum (mg L^{-1})	0.00	0.00

Table S3. Characterization of the initial study waters with and without the presence of cyanobacteria used in the tests with different cellular proportions of MA and CR species

Parameters	Cyanobacteria-free water	Cyanobacteria water
Turbidity (NTU)	38.85 ± 2.02	41.34 ± 1.66
pH	7.81 ± 0.09	7.87 ± 0.11
Color (HU)	73.33 ± 3.05	114.33 ± 6.41
Abs254nm (cm^{-1})	0.045 ± 0.002	0.039 ± 0.002
Conductivity (µS cm^{-1})	160 ± 0.040	140 ± 0.044
Residual aluminum (mg L^{-1})	0.00	0.00
Dissolved organic carbon DOC (mg L^{-1})	5.35 ± 0.60	5.62 ± 0.42
SUVA254 nm (L cm^{-1} mg^{-1})	0.841	0.781

Table S4. Cell Proportions of the Species *Microcystis aeruginosa* (MA) and *Cylindrospermopsis raciborskii* (CR) Evaluated in the Stage of Cell Proportion Influence

Cellular proportions (%)	MC Cell Density (cell mL^{-1})	CR Cell Density (cell mL^{-1})
100% MC: 0% CR	5.15 x 10^5	0.00
75% MC: 25% CR	3.76 x 10^5	1.20 x 10^5
50% MC: 50% CR	2.60 x 10^5	2.80 x 10^5
25% MC: 75% CR	1.30 x 10^5	3.80 x 10^5
0% MC: 100% CR	0.00	5.36 x 10^5

Table S5 - Residual values and efficiencies obtained for color and turbidity, before and after treatment with C/F/DAF, in the cell proportion study for cyanobacteria-free water and cyanobacteria water

Conditions	Turbidity (NTU)	Efficiency (%)	Color (HU)	Efficiency (%)
Initial cyanobacteria-free water	38.85 ± 2.00	0.0	73.33 ± 5.45	-
Post-treatment cyanobacteria-free water	4.61 ± 0.530	88.1	11.66 ± 4.36	84.0
Initial cyanobacteria water	41.34 ± 1.66	0.0	114.33 ± 6.41	-
100% MC: 0% CR	6.45 ± 0.13	84.3	33.33 ± 3.34	70.8
75% MC: 25% CR	5.85 ± 0.25	85.8	30.00 ± 3.33	73.7
50% MC: 50% CR	2.44 ± 1.75	94.0	23.33 ± 5.09	79.5
25% MC: 75% CR	4.91 ± 0.20	88.1	28.33 ± 3.85	75.2
0% MC: 100% CR	6.33 ± 0.54	84.6	30.00 ± 3.33	73.7

Figure S1 - Standard Curve for Color Quantification

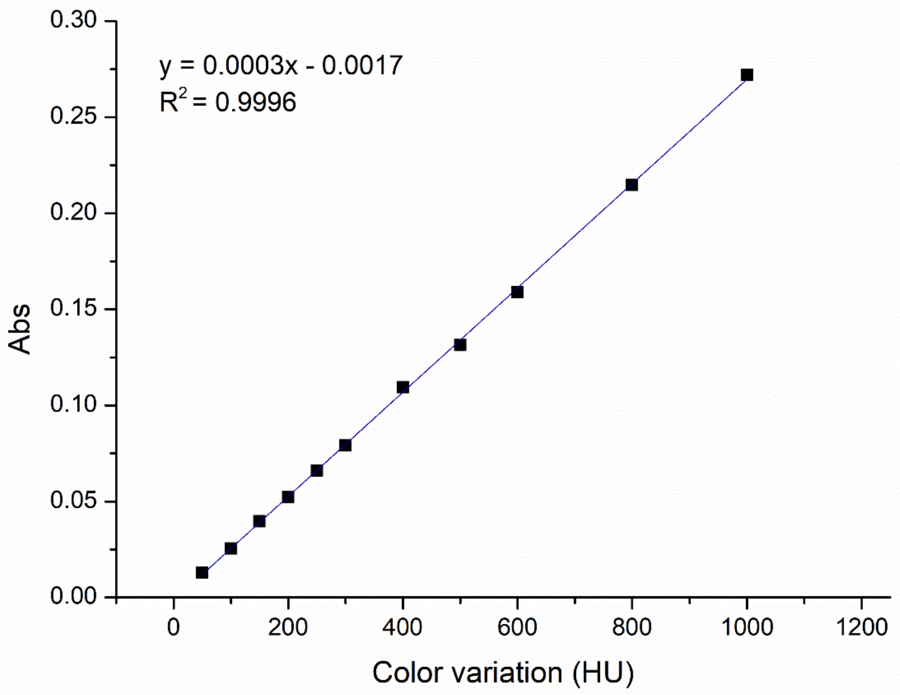

Supplement: Supplementary file 1 [file ao5c03206_si_001.pdf]
